# Comparison of Surti goat milk with cow and buffalo milk for gross composition, nitrogen distribution, and selected minerals content

**DOI:** 10.14202/vetworld.2016.710-716

**Published:** 2016-07-12

**Authors:** Dhartiben B. Kapadiya, Darshna B. Prajapati, Amit Kumar Jain, Bhavbhuti M. Mehta, Vijaykumar B. Darji, Kishorkumar D. Aparnathi

**Affiliations:** 1 Department of Dairy Chemistry, SMC College of Dairy Science, Anand Agricultural University, Anand, Gujarat, India; 2 Department of Agricultural Statistics, BA College of Agriculture, Anand Agricultural University, Anand, Gujarat, India

**Keywords:** gross composition, nitrogen distribution and mineral content, Surti goat milk

## Abstract

**Aim:**

The study was undertaken to find out the gross composition, nitrogen distribution, and selected mineral content in Surti goat milk, and its comparison was made between cow and buffalo milk.

**Materials and Methods:**

Goat milk samples of Surti breed and buffalo milk samples were collected during the period from July to January 2014 at Reproductive Biology Research Unit, Anand Agricultural University (AAU), Anand. Cow milk samples of Kankrej breed were collected from Livestock Research Station, AAU, Anand. Samples were analyzed for gross composition such as total solids (TS), fat, solid not fat (SNF), protein, lactose, and ash. Samples were also analyzed for nitrogen distribution such as total nitrogen (TN), non-casein nitrogen (NCN), non-protein nitrogen (NPN), and selected minerals content such as calcium, magnesium, phosphorous, and chloride. Total five replications were carried out.

**Results:**

Goat milk had the lowest TS, fat, protein, and lactose content among all three types of milk studied in the present investigation. On the other hand, the highest TS, fat, protein, and lactose content were found in buffalo milk. Buffalo milk had the highest SNF, calcium, magnesium, and phosphorous content, which was followed by goat milk and lowest in cow milk. The SNF, protein, TN, and calcium content of goat milk were statistically non-significant (p<0.05) with cow milk. The lactose content of goat milk was significantly lower (p>0.05) than that of the cow milk as well as buffalo milk. The goat milk had the highest ash and NCN content, which were followed by buffalo milk and lowest in cow milk. However, the differences in ash, NPN, and phosphorous content of three types of milk studied, *viz*., goat milk, cow milk, and buffalo milk were found statistically non-significant (p<0.05). The NCN content of buffalo milk was statistically non-significant (p<0.05) with cow milk as well as goat milk. The NCN and magnesium content of goat milk were significantly higher (p>0.05) than that of the cow milk. The magnesium content of goat milk was statistically non-significant (p<0.05) with buffalo milk. The chloride content of goat milk was significantly higher (p>0.05) than that of the buffalo milk as well as cow milk.

**Conclusion:**

It can be concluded from the study that the goat milk has lower TS, fat, lactose, protein content, TN as well as NPN but higher ash and NCN content compared to cow milk and buffalo milk. The goat milk has lower calcium, phosphorous compared to buffalo milk while it has higher calcium, phosphorous compared to cow milk, and it has higher magnesium, chloride content compared to cow milk and buffalo milk.

## Introduction

Milk is considered as nearly complete human food and it is considered as the first food for the newly born offspring. There are numerous studies throughout the world and thousands of references available, especially with regard to milk consumed by humans. However, the literature data mainly concern cow and buffalo milk and to a lesser extent, goat and sheep milk.

Goat milk offers a wide variety of health benefits such as better digestibility [1], more alkalinity [2], less αs1 casein than cow’s milk and is, therefore, less allergenic [3]. Goat milk also has antioxidant, antimicrobial, and medicinal property [4,5]. Goat milk contains a higher carotene (pro-vitamin A) having cancer-preventing properties. It is also useful in the treatment of ulcers due to its more effective acid buffering capacity [6]. Goat milk has a stronger flavor due to the liberation of short-chain fatty acids during rough handling, which gives off a goaty smell [7,8]. Unlike cow milk, which is slightly acidic, goat milk is alkaline in nature, which is very useful for people with acidity problems [2].

Goat milk is more digestible because of its small-sized globules, uniform protein, fat distribution, and less lactose. Modified goat milk can also be used in baby feeding [1]. Goat milk provides a healthy and a balanced diet for the children who are allergic to cow milk, as the symptoms may disappear with goat milk consumption [3]. Nutritional value of milk is closely related with its composition, which is highly affected by factors such as breed, feed, stage of lactation, and season [3,9]. Particularly during lactation, there are significant changes in the amount and composition of goat milk [10]. Various symptoms are reduced due to protein allergy, inability to digest lactose (particularly with lactose intolerance people) by taking goat’s milk.

The vitamin and mineral contents of goat’s milk and cow’s milk are fairly similar, though goat’s milk contains more calcium, vitamin B6, vitamin A, potassium, niacin, copper, and the antioxidant selenium. Goat milk is an excellent source of calcium, phosphorus, and potassium. It is also a good source of magnesium, sodium, and iron [3,11]. Calcium helps to protect against colon cancer, improves blood clotting ability, and helps to maintain healthy blood pressure and to prevent muscle cramps/contractions. Goat milk is a good source of calcium, containing approximately 13% more calcium than cow’s milk. Magnesium is particularly beneficial to the heart, helping to maintain a regular heartbeat, preventing the formation of blood clots and raising good cholesterol levels. It also works with calcium and vitamin D to maintain healthy bones. Goat milk has a higher content of magnesium than cow’s milk. Phosphorus works in conjunction with calcium and vitamin D that can not only build and maintain strong bones but also plays a role in activities of the brain, kidney, heart, and blood. Goat milk has a higher phosphorous content than cow’s milk [12,13]. The value of goat milk in human nutrition has so far received very little academic attention.

Estimated population of Milch goat for the Gujarat state during the year 2010-2011 was estimated at 27,181 hundred, which was 0.63% increased over previous year’s estimate (during 2009-2010) of 27,011 hundred. The total estimated milk production of goat for the state during 2010-2011 works out to be 235.61 thousand tons, which was increased 1.89% over the previous year’s estimate (during 2009-2010) of 231.24 thousand tons. Estimated number of productive goats in Anand during 2010-2011 was 374 hundred [14].

The Food Safety and Standards Authority of India (FSSAI), Ministry of Health and Family Welfare, and Government of India were interested to develop new standards of milk and required to generate a standard database on milk and milk products. The data on gross composition, nitrogen distribution, mineral content, and related information of milk of various species and breed of animals were lacking. Moreover, there were no reported values on these aspects of goat milk in Anand/Gujarat. Therefore, there was a need to undertake a systematic study to generate data. This work provides basic database for gross composition and nitrogen distribution pattern and selected mineral content of milk of Surti goat. This information will be beneficial to goat keepers, industrial personnel, and various government agencies as well as to society.

## Materials and Methods

### Ethical approval

Ethical approval was not applicable for this study. However milk samples were collected as per standard milking techniques.

### Milk samples

Goat milk samples of Surti breed and buffalo milk samples were collected during the period from July to January 2014, at Reproductive Biology Research Unit, Anand Agricultural University (AAU), Anand. Cow milk samples of Kankrej breed were collected from Livestock Research Station, AAU, Anand, and used for investigation. Raw milk samples were collected at milking time in a clean and dry container. The samples were transported to the laboratory. Total five replications were carried out.

### Chemical and glassware

During the entire study, chemicals used for chemical analysis for various properties were of analytical grade.

### Gross chemical composition of milk

Total solids (TSs) in all the milk samples were determined gravimetrically by the procedure as described in Bureau of Indian Standards (BIS) handbook [15]. The milk fat content in all the milk samples was estimated by following the Gerber method as described in BIS handbook [15]. Solid not fat (SNF) content of goat, cow, and buffalo milk was calculated by subtracting percent fat content of milk samples from their respective percent TS content of the milk. The protein content of all the milk samples was determined using the micro Kjeldahl method of nitrogen estimation as described in BIS Handbook [15]. The percent total protein was obtained by multiplying the percent nitrogen by a factor of 6.38. The lactose content of all the milk samples was determined using Lane-Eyon Method described in BIS handbook [15]. The procedure outlined in BIS handbook [15] was followed for determination of ash content in all the milk samples.

### Nitrogen distribution

Total nitrogen (TN) content of all the milk samples was determined using the micro Kjeldahl method of nitrogen estimation as described in BIS handbook [15]. Non-casein nitrogen (NCN) content and non-protein content of all the milk samples were determined using Rowland’s analytical scheme for nitrogen fractions of milk as described in a laboratory manual on chemical analysis of milk protein by Kumar *et al*. [16].

### Selected minerals content

Calcium and magnesium were determined simultaneously in milk by complexometric method of Davies and White [17] using disodium salt of ethylenediaminetetraacetic acid. Phosphorous was determined by the colorimetric method of Fiske and Subbarow [18]. The chloride content of all the milk sample of milk was determined by Hammer and Bailey [19] using AgNO_3_.

### Statistical analysis

The collected data were subjected to statistical analysis. Data were analyzed by completely randomized design and critical difference test at 5% level of significance (p<0.05) as per the procedure mentioned by Steel and Torrie [20].

## Results

The gross composition of goat, cow, and buffalo milk is mentioned in Table-1. The range of TS content in goat milk was 12.32-13.02% with a mean value of 12.76%. Similarly, in cow milk, range of TS was 13.29-14.20% with a mean value of 13.79%. On the other hand, TS content ranged between 17.88% and 19.18% with a mean value of 18.45% in buffalo milk. Thus, TS content of goat milk was significantly lower (p>0.05) than that of the cow milk as well as buffalo milk. The range of fat content in goat milk was 3.4-4.2% with a mean value of 3.84%. Similarly, in cow milk, range of fat was 4.2-5.5% with a mean value of 4.88%. On the other hand, fat content ranged between 7.9% and 8.8% with a mean value of 8.30% in buffalo milk. The fat content of goat milk was significantly lower (p>0.05) than that of the cow milk as well as buffalo milk. The range of SNF content in goat milk was 8.37-8.82% with a mean value of 8.57%. Similarly, in cow milk, range of SNF was measured to be 8.37-8.83% with a mean value of 8.54%. On the other hand, SNF content ranged between 9.04% and 9.68% with a mean value of 9.48% in buffalo milk. The SNF content of goat milk was statistically non-significant (p<0.05) with cow milk. The range of protein content in goat milk was 3.18-3.49% with a mean value of 3.42%. Similarly, in cow milk, range of protein was 3.19-3.62% with a mean value of 3.49%. On the other hand, protein content ranged between 4.11% and 4.74% with a mean value of 4.48% in buffalo milk. The protein content of goat milk was statistically non-significant (p<0.05) with cow milk. The range of lactose content was 3.78-4.45% with a mean value of 4.16% in goat milk. Similarly, in cow milk, range of lactose was 4.45-5.31% with a mean value of 4.76%. On the other hand, lactose content ranged between 4.67% and 5.27% with a mean value of 4.86% in buffalo milk. The lactose content of goat milk was significantly lower (p>0.05) than that of the cow milk as well as buffalo milk. The range of ash content was 0.76-1.06% with a mean value of 0.89% in goat milk. Similarly, in cow milk, range of ash was 0.73-0.79% with a mean value of 0.76%. On the other hand, ash content ranged between 0.69% and 0.89% with mean value of 0.81% in buffalo milk. The goat milk had the highest ash content, which was followed by buffalo milk and cow milk had the lowest ash content.

**Table-1:** Gross composition of goat, cow, and buffalo milk.

Types of milk	Parameters (%)					

	Fat	SNF	TS	Protein	Lactose	Ash
Goat	3.84±0.37^a^ (3.4-4.2)	8.57±0.18^a^ (8.37-8.82)	12.76±0.29^a^ (12.32-13.02)	3.42±0.14^a^ (3.18-3.49)	4.16±0.31^a^ (3.78-4.45)	0.89±0.12^a^ (0.76-1.06)
Cow	4.88±0.53^b^ (4.2-5.5)	8.54±0.17^a^ (8.37-8.83)	13.79±0.36^b^ (13.29-14.20)	3.49±0.18^a^ (3.19-3.62)	4.76±0.35^b^ (4.45-5.31)	0.76±0.02^a^ (0.73-0.79)
Buffalo	8.30±0.37^c^ (7.9-8.8)	9.48±0.25^b^ (9.04-9.68)	18.45±0.56^c^ (17.88-19.18)	4.48±0.29^b^ (4.11-4.74)	4.86±0.24^b^ (4.67-5.27)	0.81±0.08^a^ (0.69-0.89)
SEM	0.19	0.09	0.19	0.09	0.14	0.04
CD	0.59	0.28	0.58	0.29	0.42	-
Test	*	*	*	*	*	NS
CV %	7.51	2.31	2.79	5.60	6.60	10.14

a-c Values with different letters within a column are significantly different at 5% level of significant (i.e., p<0.05). SEM=Standard error of mean, CD=Critical difference, CV=Coefficient of variance, NS=Not significant, SNF=Solid not fat, TS=Total solids

The nitrogen distribution of goat, cow, and buffalo milk is mentioned in Table-2. The range of TN content was 0.498-0.548% with a mean value of 0.536% in goat milk. Similarly, in cow milk, range of TN was 0.499-0.568% with a mean value of 0.547%. On the other hand, TN ranged between 0.644% and 0.743% with a mean value of 0.702% in buffalo milk. The TN content of buffalo milk was significantly higher (p>0.05) than that of the cow milk and goat milk. The range of NCN content determined was 0.132-0.167% with a mean value of 0.153% in goat milk. Similarly, in cow milk, range of NCN was 0.103-0.136% with a mean value of 0.124%. On the other hand, NCN ranged between 0.118% and 0.157% with a mean value of 0.140% in buffalo milk. The NCN content of buffalo milk was statistically non-significant (p<0.05) with cow milk as well as goat milk. The range of non-protein nitrogen (NPN) content was 0.019-0.036% with a mean value of 0.029% in goat milk. Similarly, in cow milk, range of NPN was 0.036-0.074% with a mean value of 0.054%. On the other hand, NPN ranged between 0.023% and 0.082% with a mean value of 0.051% in buffalo milk. The differences in NPN content of three types of milk studied, *viz*., goat milk, cow milk, and buffalo milk were found statistically non-significant (p<0.05).

**Table-2:** Nitrogen distribution of goat, cow, and buffalo milk.

Types of milk	Parameters (%)		

	TN	NCN	NPM
Goat	0.536±0.021^a^ (0.498-0.548)	0.153±0.015^a^ (0.132-0.167)	0.029±0.007^a^ (0.019-0.036)
Cow	0.547±0.028^a^ (0.499-0.568)	0.124±0.013^b^ (0.103-0.136)	0.054±0.014^a^ (0.036-0.074)
Buffalo	0.702±0.046^b^ (0.644-0.743)	0.140±0.020^c^ (0.118-0.157)	0.051±0.024^a^ (0.023-0.082)
SEM	0.015	0.007	0.007
CD	0.05	0.022	-
Test	*	*	NS
CV%	5.62	11.64	37.003

a-c Values with different letters within a column are significantly different at 5% level of significant (i.e., p<0.05). SEM=Standard error of mean, CD=Critical difference, CV=Coefficient of variance, NS=Not significant, TN=Total nitrogen, NCN=Noncasein nitrogen, NPM=Nonprotein nitrogen

The selected mineral content of goat, cow, and buffalo milk is mentioned in Table-3. The calcium content determined in five replications ranged between 117.88 and 141.46 mg/100 ml with a mean value of 129.08 mg/100 ml in goat milk. Similarly, in cow milk, range of calcium was 111.99-129.67 mg/100 ml with a mean value of 120.24 mg/100 ml. On the other hand, calcium content ranged between 167.98 and 185.66 mg/100 ml with a mean value of 178.59 mg/100 ml in buffalo milk. The calcium content of buffalo milk was significantly higher (p>0.05) than that of the goat milk as well as cow milk. The magnesium content was determined between 18.48 and 21.16 mg/100 ml with a mean value of 19.94 mg/100 ml in goat milk. Similarly, in cow milk, range of magnesium was 9.73-16.54 mg/100 ml with a mean value of 12.65 mg/100 ml. On the other hand, magnesium content ranged between 14.59 and 21.40 mg/100 ml with a mean value of 18.29 mg/100 ml in buffalo milk. The magnesium content of goat milk was significantly higher (p>0.05) than that of the cow milk. The magnesium content of goat milk was statistically non-significant (p<0.05) with buffalo milk. The range of phosphorous content was 75.85-126.19 mg/100 ml with a mean value of 98.91 mg/100 ml in goat milk. Similarly, in cow milk, range of phosphorous was 78.57-97.64 mg/100 ml with a mean value of 88.08 mg/100 ml. On the other hand, phosphorous ranged between 95.22 and 124.72 mg/100 ml with a mean value of 109.22 mg/100 ml in buffalo milk. The differences in phosphorous content of three types of milk studied were found statistically non-significant (p<0.05). The chloride content was between 0.15 and 0.17% with a mean value of 0.16% in goat milk. Similarly, in cow milk, range of chloride was 0.11-0.14% with a mean value of 0.13%. On the other hand, chloride content ranged between 0.10% and 0.12% with a mean value of 0.11% in buffalo milk. The chloride content of goat milk was significantly higher (p>0.05) than that of the buffalo milk as well as cow milk.

**Table-3:** Selected mineral content of goat, cow, and buffalo milk.

Types of milk	Parameters			

	Calcium (mg/100 ml)	Magnesium (mg/100 ml)	Phosphorous (mg/100 ml)	Chloride (%)
Goat	129.08±9.41^a^ (117.88-141.46)	19.94±1.02^a^ (18.48-21.16)	98.91±19.89^a^ (75.85-126.19)	0.16±0.01^a^ (0.15-0.17)
Cow	120.24±7.04^a^ (111.99-129.67)	12.65±2.84^b^ (9.73-16.54)	88.08±7.62^a^ (78.57-97.64)	0.13±0.01^b^ (0.11-0.14)
Buffalo	178.59±6.79^b^ (167.98-185.66)	18.29±2.52^a^ (14.59-21.40)	109.22±12.16^a^ (95.22-124.72)	0.11±0.06^c^ (0.10-0.12)
SEM	3.50	1.01	6.33	0.003
CD	10.80	3.12	-	0.011
Test	*	*	NS	*
CV%	5.49	13.37	14.34	5.880

a-c Values with different letters within a column are significantly different at 5% level of significant (i.e., p<0.05). SEM=Standard error of mean, CD=Critical difference, CV=Coefficient of variance, NS=Not significant

## Discussion

Goat milk has health beneficial properties. The information on composition, nitrogen distribution, and selected mineral content of Surti goat milk produce in Gujarat is not available. Moreover, there is a need to take systematic study on comparative analysis of goat, cow, and buffalo milk for their composition, nitrogen distribution, and selected minerals content.

Shettar [21] reviewed that the TS content of Marwari, Assam local breed, Beetal × Assam local crossbred, and Jakhrana goats as 14.57%, 15.77%, 14.92%, and 13.55%, respectively. Soliman [22] analyzed goat milk and found that it contained 12.62% TS. Mahmood and Usman [12] reported that TS content of goat milk was 12.84%. TSs content in milk of various breeds of cow such as Ayrshire, Brown Swiss, Guernsey, Holstein, Jersey, and Zebu was 13.1%, 13.3%, 14.4%, 12.2%, 15.0%, and 14.7% and fat content in the same was 4.1%, 4.0%, 5.0%, 3.5%, 5.5%, and 4.9%, respectively [23]. The average TSs and fat content ranged between 16.39-18.48% and 6.57-7.97% in buffalo milk [24]. The data obtained in present study for average percent TS content of goat milk were lower than reported in the literature for milk obtained from goat in India. Similarly, the data obtained for average TS content of cow and buffalo milk were also in general agreement with those reported in the literature for cow and buffalo milk, respectively. Arora *et al*. [25] analyzed Indian goat milk and found 3.73% fat, 3.02% protein, and 4.45% lactose. Shettar [21] reviewed that the milk of Jakhrana goats had 8.60±0.10% SNF. Mahmood and Usman [12] reported the fat content of goat milk as 3.97%. Park *et al*. [26] reported 9.0% SNF, 3.6% fat, and 0.7% ash in cow milk. Protein and lactose content in milk of various breeds of cow such as Ayrshire, Brown Swiss, Guernsey, Holstein, Jersey, and Zebu were found as 3.6%, 3.6%, 3.8%, 3.1%, 3.9%, and 3.9% and 4.7%, 5.0%, 4.9%, 4.9%, 4.9%, and 5.1%, respectively [23]. Rao and Nagarcenkar [27] reviewed average SNF, protein, lactose, and ash percent in Murrah (Indian) milk were 10.2%, 4.5%, 5.1%, and 0.8%, respectively. Sachdeva *et al*. [28] found that the milk of Barbari and Jamnapari goat milk had ash 0.82% and 0.9%. Hanl *et al*. [24] reported range of fat content between 6.57% and 7.97% in buffalo milk. Rao and Nagarcenkar [27] reviewed average protein and SNF percent in the milk of Sichun buffaloes. The values reported were 5.2% and 10.0%, respectively. The data obtained in the present study for average percent fat, SNF, protein, and ash content of goat milk were in general agreement with those reported in the literature for milk obtained from goat in India. The data were also in general agreement with those reported for goat milk obtained from exotic goat. Similarly, the data obtained for average percent ash content of cow and buffalo milk were also in general agreement with those reported in the literature for cow and buffalo milk, respectively.

Milk from Beetal goats contained less TN and NCN than milk from the Jamnapari, Barbari, and Bengal breeds (TN 0.518 vs. 0.532-0.534% and NCN 0.122 vs. 0.125-0.128%) [29]. Jenness [30] analyzed six samples from pygmy goats and reported average nitrogen distribution as 0.840% TN, 0.182% NCN, and 0.053% NPN. The reported range from literature about nitrogen distribution in pooled cow milk for TN was 0.43-0.70% [11]. Li *et al*. [31] studied nitrogen distribution of cow milk and reported 0.08-0.16% NCN and 0.023-0.042% NPN in cow milk. The distribution of nitrogen in different fractions of pooled Indian buffalo milk was studied by Sindhu and Singhal [32] and results obtained for TN, NCN, and NPN were 0.600%, 0.140%, and 0.035%, respectively. The data obtained in the present study for average percent TN and NCN content of goat milk were in general agreement with those reported in the literature for milk obtained from goat in India. The data were also in general agreement with those reported for goat milk obtained from exotic goat. Similarly, the data obtained for average percent TN and NCN content of cow and buffalo milk were also in general agreement with those reported in the literature for cow and buffalo milk, respectively. The data obtained in the present study for average percent NPN content of goat milk were in general agreement with those reported in the literature for goat milk. The data obtained for average percent NPN content of cow and buffalo milk were slightly higher in general agreement with those reported in the literature for cow and buffalo milk, respectively. In the literature, percent NPN content in goat milk is reportedly higher than that in buffalo milk as well as cow milk. However, in the present study, goat milk was found to contain lower NPN compared to buffalo milk as well as cow milk. The comparative composition of proteins and their components in the milk of goats and cows have been reviewed by Jenness [33] and Haenlein [34], identifying many unique differences between the two species, and showing a wide diversity due to genetics of different breeds within each species, influences of stage of lactation, feeding, climate, and subclinical mastitis. Goat milk proteins are similar to the major cow milk proteins in their general classifications of α-, β-, κ-caseins, β-lactoglobulin, α-lactalbumin, but they differ widely in genetic polymorphisms and their frequencies in goat populations [35].

Soliman [22] studied goat milk and found the average value of calcium as 130.28 mg/100 g. Pal *et al*. [36] reported the magnesium and phosphorous content of goat milk as 20 mg/100 g and 130 mg/100 g. Pal *et al*. [36] reported calcium, magnesium, and phosphorous content of cow milk as 120 mg/100 g, 12 mg/100 g, and 95 mg/100 g. Soliman [22] studied cow milk and found the average value of calcium as 119.90 mg/100 g and average value of magnesium as 13.42 mg/100 g. The average calcium content of buffalo milk was reported to be 180 mg/100 g [37]. Magnesium and phosphorous content of buffalo milk ranged between 16 and 30 mg/100 g and 89-137 mg/100 g, respectively [38,39]. The data obtained in the present study for average calcium, magnesium, and phosphorous content of goat milk were in general agreement with reported in the literature for milk obtained from goat. Similarly, the data obtained for average calcium, magnesium, and phosphorous content of cow and buffalo milk were also in general agreement with reported in the literature for cow and buffalo milk, respectively. Shettar [21] reviewed chloride content in the milk of Marwari goat as 0.24±0.02%. The average chloride content of goat milk and cow milk were reported as 150 mg/100 g and 100 mg/100 g [26]. The chloride content of buffalo milk was 57-106 mg/100 g [19,38]. The data obtained in the present study for average percent chloride content of goat milk were in general agreement with those reported in the literature for goat milk. Similarly, the data obtained for average percent chloride content of cow and buffalo milk were higher than with those reported in the literature for cow and buffalo milk, respectively.

## Conclusion

Although goat milk has more health beneficial properties, the data on indigenous goat milk are very scanty and fragmented. Moreover, FSSAI is interested to develop new standards database on milk and milk products of various species and breed of animals. There were no reported values on these aspects of Surti goat milk as well as no comparison was made so far with that of cow and buffalo milk in Anand/Gujarat. It can be concluded from the study that the Surti goat milk has lower TS, fat, lactose, protein, TN, and NPN but higher ash as well as NCN content compared to cow milk and buffalo milk. The goat milk has lower calcium, phosphorous compared to buffalo milk while it has higher calcium, phosphorous compared to cow milk, and it has higher magnesium, chloride content compared to cow milk and buffalo milk.

## Authors’ Contributions

KDA supervised the experiment. DBK conducted the experiment and the laboratory analysis of the samples. DBK along with AKJ analyzed the data. DBK and BMM prepared the manuscript. VBD has helped in statistical analysis of the data. DBK, AKJ, BMM, DBP, and KDA reviewed the manuscript. All authors read and approved the final manuscript.
